# Increase in Milk Yield from Cows through Improvement of Forage Production Using the N_2_-Fixing Legume *Leucaena leucocephala* in a Silvopastoral System

**DOI:** 10.3390/ani10040734

**Published:** 2020-04-23

**Authors:** Lucero Sarabia-Salgado, Francisco Solorio-Sánchez, Luis Ramírez-Avilés, Bruno José Rodrigues Alves, Juan Ku-Vera, Carlos Aguilar-Pérez, Segundo Urquiaga, Robert Michael Boddey

**Affiliations:** 1 Department of Soils, Federal Rural University of Rio de Janeiro (UFRRJ), Seropédica, Rio de Janeiro 23897-000, Brazil; 2 Faculty of Veterinary Medicine and Animal Science, Autonomous University of Yucatán, Mérida, Yucatán 97315, Mexico; ssolorio@correo.uady.mx (F.S.-S.); luis.ramirez@correo.uady.mx (L.R.-A.); kvera@correo.uady.mx (J.K.-V.); caperez@correo.uady.mx (C.A.-P.); 3 Embrapa Agrobiologia, Brazilian Corporation for Agricultural Research—National Centre for Agrobiology Research, Seropédica, Rio de Janeiro 23891-000, Brazil; bruno.alves@embrapa.br (B.J.R.A.); segundo.urquiaga@embrapa.br (S.U.); robert.boddey@embrapa.br (R.M.B.)

**Keywords:** climate change, dry season, livestock-system, ^15^N, N_2_-fixation

## Abstract

**Simple Summary:**

In tropical livestock production, forage availability and quality are a serious constraint for milk and meat production. There is an urgent need to reduce the environmental impact of animal production while increasing productivity. The use of legume trees or shrubs associated with grasses effectively increased milk production and decreased the need to use nitrogen fertilizers by taking advantage of atmospheric nitrogen fixation.

**Abstract:**

The objective was to evaluate milk production, N_2_-fixation and N transfer, forage yield and composition (under two cutting intervals) in a silvopastoral system (SPS) with *Leucaena leucocephala-Megathyrsus maximus* and *M. maximus*-monoculture (MMM) with crossbred cows in a completely randomized design. Forage yield in the SPS was 6490 and 6907 kg DM ha^−1^ for cutting intervals (CI) of 35 and 50 days. Forage yield for the MMM was 7284 and 10,843 kg DM ha^−1^, and forage crude protein (CP) was 29.0% and 26.1% for *L. leucocephala*, harvested at 35 and 50 days, respectively. CP for the associated *M. maximus* was 9.9% and 7.8% for CI 35 and 50 days, respectively, and for MMM was 7.4% and 8.4%, harvested at 35 and 50 days. Milk production was 4.7 kg cow^−1^ day^−1^ for cows grazing MMM and 7.4 kg cow^−1^ day^−1^ under SPS. Nitrogen fixation in *L. leucocephala* (%Ndfa) was estimated to be 89% and 95%, at 35 and 50 days, with an N_2_ transfer to the associated grass of 34.3% and 52.9%. SPS has the potential to fix and transfer important amounts of N_2_ to the associated grass, and increase forage CP content and milk production.

## 1. Introduction

Cattle production in tropical regions of the world face tremendous constraints, including inadequate management, poor quality and availability of forage resources and, ultimately, the impacts of climate change. Among other reasons, low soil fertility and the fluctuations in rainfall have caused the majority of pastures to deteriorate. Pastures now generally require large quantities of chemical fertilizers, especially nitrogen, in order to maintain forage production throughout the year. However, fertilizers are costly and can have a large impact on surrounding ecosystems [1]. The application of fertilizers to pastures promotes the production of nitrous oxide in soil, a greenhouse gas with 265 times the global warming potential relative to carbon dioxide [2]. Even considering that N fertilization improves forage quality, tropical pastures contain low crude protein (CP) and high neutral detergent fiber (NDF) during the dry season and when consumed, they are important contributors to greenhouse gas (GHG) emissions, especially methane from enteric processes by cattle [3]. 

Silvopastoral systems (SPS) have been proposed as a sustainable strategy to solve the problem provoked by grazing systems based on grass monocultures. These systems can integrate both legume trees and grasses in order to increase the production and quality of forage while providing extra N to the system from biological N_2_ fixation [4,5]. Leguminous tree species generally produce a richer protein fodder during the wet season, bringing about a higher stocking rate, while their deeper root systems allow the offer of green forage to cattle during the dry season [6]. For tropical regions, the legume *Leucaena leucocephala* (Lam.) De Wit., frequently referred to as leucaena, has been considered as a protein bank or even as a pasture component. 

Even though the reduction in costs associated with nitrogen fertilization and the perspective of the low GHG emission intensity of legume-based SPS have been demonstrated [7], some of their components and management under field conditions have not been fully evaluated. This is the case insofar as the question of cutting intervals is concerned, which is an important management factor associated with total biomass production and the transfer of atmospheric nitrogen. 

The objective of this research was to evaluate the effect on milk yield of crossbred cows grazing either a Tanzania grass (*Megathyrsus maximus* (Jacq.) B.K. Simon and S.W.L. Jacobs) pasture under N fertilization or in a SPS with *Leucaena leucocephala*. In addition, this study aimed to evaluate the effect of two fodder cutting intervals (35 and 50 days) on dry matter production and the quality of forage and the contribution of N_2_ fixation to the SPS.

## 2. Materials and Methods 

### 2.1. Area Location and Characteristics

**Figure 1:**
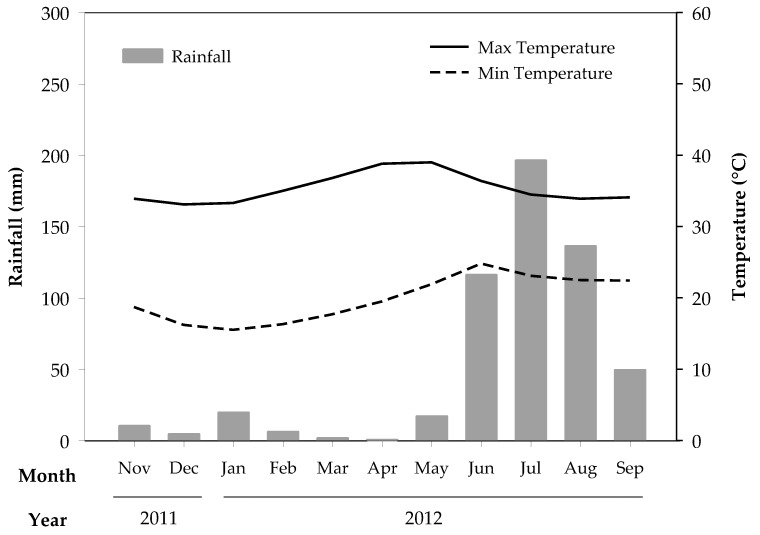
Monthly meteorological data for the experimental period (November 2011 to September 2012).

The study was carried out from December 2011 to September 2012, in the municipality of Apatzingán in the state of Michoacán, Mexico, at 19° 05’ N and 102° 21’ W and 325 m a.s.l. The soil is classified as a Vertisol (19% sand, 17% silt and 64% clay) according to FAO classification, with a pH of 8.1, 22.5 g organic matter kg^−1^, 5.8 mg P kg^−1^, 585 mg K kg^−1^ and 9356 mg Ca kg^−1^, all quantified according to Anderson and Ingram (1993) [8]. The climate is hot semi-arid with a mean annual precipitation of 924 mm and an average mean temperature of 28 °C [9]. The rainy season is from May to October, but rains are not completely absent in the remaining months. For the experimental period, the rainfall and temperature regimes are displayed in Figure 1.

### 2.2. Characterization and Management of the Studied Area

Sixty-six multiparous crossbred cows (*Bos taurus* × *Bos indicus*) of 480 ± 20 kg live weight and a mean of 50 days in milk were used in a completely randomized design. Cows were divided into homogenous groups based on their milk yield and assigned to two pasture types, which were the experimental treatments. A control group (n = 33 cows) was grazing a traditional monoculture (grass pasture-only) of Tanzania grass (*Megathyrsus maximus*) that has been fertilized with 100 kg N ha^−1^ split in three doses of approximately 70 kg urea ha^−1^, which was applied every four months starting on 15 January (treatment denominated MMM). The second treatment was the silvopastoral system (n = 33 cows), formed by the *L. leucocephala* + *M. maximus* association (SPS). Both pastures had been established for two years when Leucaena was planted in SPS in rows in an N–S direction with 1.6 m between rows and 0.3 m between trees within a row, representing 30% of the SPS area. The MMM occupied 30 ha with a stocking rate of 1.1 cab ha^−1^, while the SPS was carried out on 18 ha with 1.3 cab ha^−1^. The pasture management in both treatments was rotational grazing, where cows were allowed to graze for two days followed by 33 days resting. The area of each paddock was, on average, 5000–6000 m^2^ in both systems with a grazing time from 13:00 to 05:00 h for both groups of cows, which were sent to milking once a day at 05:00 a.m. Milking was mechanical and milk yield was measured at each milking time during two weeks each month, from December, 2011 to August, 2012. The cows used in the study were in milking condition until the end of the monitoring time. In the case of any dry cows, they was replaced by another cow with the same lactation stage as the group cows under study. The average milk production of the five working days from each week were used for the statistical analysis. Just before milking, calves were allowed to suckle to promote milk-down. In the time after milking, the cows were exposed to the calves to suckle the residual milk until they returned to the pastures. The described management is typical of the region where milk production occurs year-round, but peaking in summer.

The evaluation of forage production in both systems was carried out as follows: two forage cutting intervals (CI) were evaluated to 35 and 50 days, over a period of 105 and 100 days, respectively. For the frequency of 35 days, three harvests were made and for the frequency of 50 days, two harvests. The impact of the change from a 35-day CI to a 50-day CI on forage yield and quality was studied in a neighboring experiment with 4.5 ha of SPS (28,000 Leucaena plants ha^−1^) and 0.5 ha of MMM following the same management as described for the larger area. Each area was divided into six paddocks and within each paddock; in order to evaluate the forage amount and utilization, two exclusion cages (2 × 1.5 m) were installed for the 35-day and 50-day CI treatments, respectively. On June, 16th 2012, the grass within the cages was cut to 15 cm height, while the leucaena was pruned to 50 cm, and all the residue generated was removed. From this date, the growing forage was cut at 35-day or 50-day intervals and the legume and grass material were separated. The legume was separated into edible forage (leaves and stems less than 5 mm in diameter) and non-edible woody stems. For both legume and grass, three samples were taken from each exclusion area and the fresh weight recorded. After obtaining fresh weight, approximately 300 g of this fresh matter was oven dried at 65 °C until reaching constant weight to determine dry matter.

### 2.3. Forage Chemical Composition

Samples of *L. leucocephala* and *M. maximus* were prepared and analyzed to determine the dry matter (DM) following the procedure described in [10]. Total N was determined with a CN-2000 series 3740 Leco (Leco Corporation) elemental analyzer, total nitrogen determination (crude protein % = Total N % × 6.25). Neutral detergent fiber (NDF) was determined in accordance with the method described by [11]. 

### 2.4. Fixation and Transfer of Atmospheric Nitrogen (N_2_) 

The contribution of biological nitrogen fixation (BNF) to the legume trees was assessed by the ^15^N natural abundance method [12]. Forage samples of *L. leucocephala* taken from the same cut material every 35 or 50 days were dried and finely milled (<200 mesh). The analysis of ^15^N natural abundance was performed with a Finnigan continuous-flow isotope-ratio mass spectrometer, model Delta Plus, in the “John Day Stable Isotope Laboratory” of Embrapa Agrobiologia, Seropédica, RJ, Brazil. The mass spectrometer releases a direct readout in δ^15^N units, and the software calculates this from the equation

δ^15^N (‰) = [(Rsample – Rstandard)/Rstandard ] × 1000 (1)

where R is the ratio of ^15^N/^14^N atoms in the sample and standard. The standard used is atmospheric nitrogen which is defined as 0.00‰. Secondary standards of known ^15^N abundance were used to calibrate the instrument.

The formula used to estimate the N derived from the air via BNF (%Ndfa) was applied [13]

%Ndfa = 100 (δ^15^N ref − δ^15^Nfixing plant)/(δ^15^Nref − B) (2)

where δ^15^Nref is the value of δ^15^N from plant-available N obtained from a reference species that does not fix nitrogen.

δ^15^Nfixing plant is the value of δ^15^N from the legume, *L. leucocephala*. The *B*-value (i.e., the isotopic fractionation of ^15^N between aerial and belowground tissues) was determined by growing plants of *L. leucocephala* in N-free culture.

With the results of %Ndfa, the quantity of fixed nitrogen in the system was calculated with the following formula [14]:

Amount of fixed N_2_ = (%Ndfa/100) × (total N in plant) (3)

### 2.5. Design and Statistical Analysis

A completely random design with three repetitions was used (n = 3), with factorial arrangement 2 × 2 (two systems × two defoliation frequencies). The data were analyzed using repeated measures analysis (rmMANOVA) with SigmaPlot version 11.0.

## 3. Results

### 3.1. Milk Production

**Figure 2:**
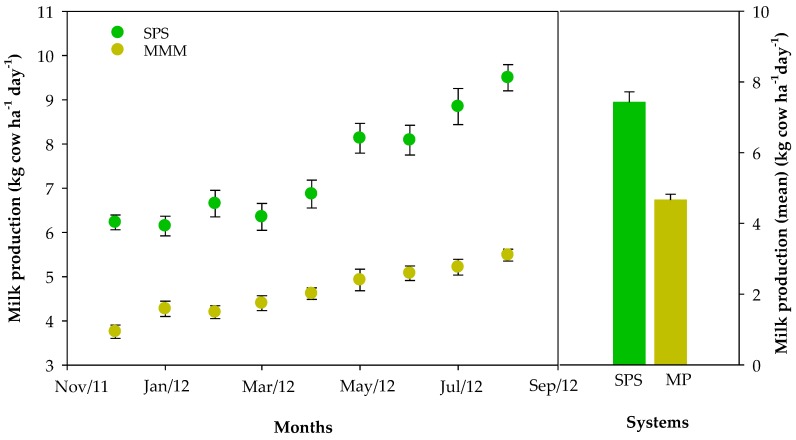
Milk production (kg cow ha^−^^1^ day^−^^1^) in the Silvopastoral System (SPS) and the *Megathyrsus maximus* in Monoculture (MMM).

The lowest values of milk production were in the dry season, with a mean of 3.8 kg cow^−1^ day^−1^ for MMM in December and 6.1 kg cow^−1^ day^−1^ for SPS in January. In August in the middle of the rainy season, yields reached 5.5 and 9.5 kg cow^−1^ day^−1^ for MMM and SPS, respectively (Figure 2). During the whole 9-month period (December 2011 to August 2012), milk yields were significantly higher (*p* < 0.001) in the SPS compared to the MMM for every individual monthly estimate. Average milk yields were 4.7 and 7.4 kg cow^−1^ day^−1^ and this difference was highly significant (*p* < 0.001).

### 3.2. Forage Yield

**Figure 3:**
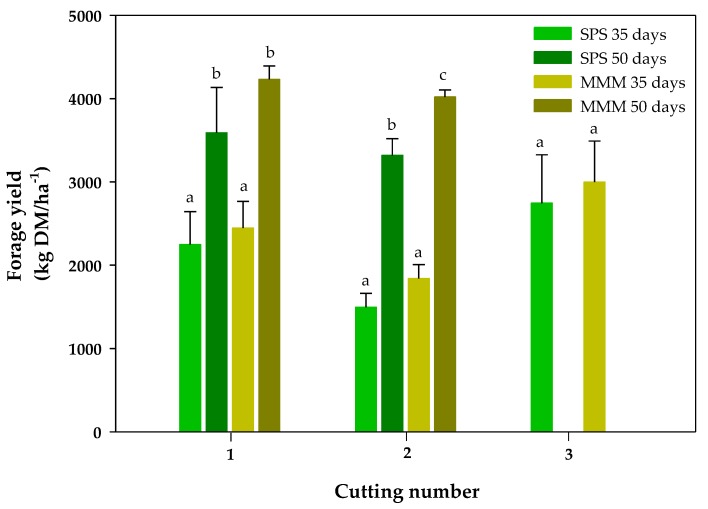
Total forage yield (kg DM ha^−^^1^) in the Silvopastoral System (SPS) and the *Megathyrsus maximus* in Monoculture (MMM) under two cutting intervals of 35 and 50 days.

**Figure 4:**
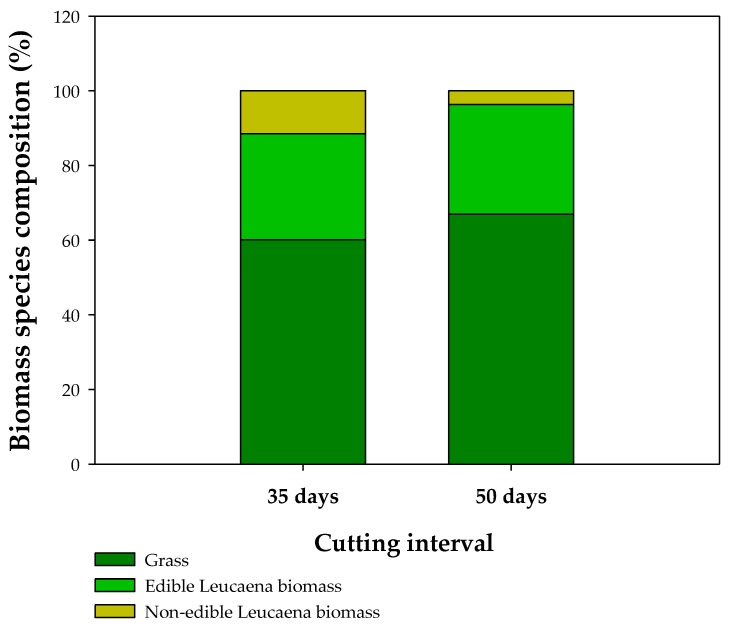
Biomass species composition (%) from a SPS based on *L. leucocephala* and *M. maximus.*

The forage yield (dry matter availability) at the first cutting/pruning was greater after the growth interval of 50 days (50-day CI) in both experimental systems (SPS and MMM), as would be expected for the longer growth period (Figure 3). The same trend was observed at the second cutting/pruning event, but growth periods of 35 days and 50 days were much less coincidental. Another exception was the greater forage yield of MMM in comparison to SPS for the 50-day CI. Only the 35-day CI had a third cutting/pruning, in which SPS and MMM presented the same dry mass availability. To compare the forage yields between the two cutting intervals, the yields of the three 35-day CI and of the two 50-day CI were summed up. In SPS, 29% of the available dry matter accumulated after 105 days corresponded to the legume in the 35-day CI treatment, practically the same as was observed after 100 days (28%) in the 50-day CI (Table 1). Nonetheless, the proportion of edible legume in the botanical composition was higher (31%) for the forage harvested in the 35-day CI than in the 50-day CI (Figure 4).

Both SPS and MMM in the 35-day CI and SPS in the 50-day CI accumulated similar dry mass, ranging from 6.4 to 6.9 Mg ha^−1^, but significantly below the 10.7 Mg ha^−1^ of dry mass accumulation in MMM after 100 days in the 50-day CI (Table 1).

On the other hand, total N accumulation by MMM in the 35-day CI was significantly lower than in the other treatments. The two SPS and MMM in the 50-day CI treatments accumulated, on average, 60% more N than MMM in the 35-day CI (Table 1). Obviously, differences in chemical composition between legume and grass tissues is an explanation, which is even more important when edible components are assessed.

### 3.3. Forage Chemical Composition

Comparing the two plant species, the level of crude protein in the legume was approximately three times that of the grass and the levels of fiber were approximately half (Table 2). Crude protein values (CP) from *L. leucocephala* (SPS) in the 35-day CI treatment were significantly higher in comparison to 50-day CI treatment (29.0% vs. 26.1%, respectively). For *M. maximus* forage (in SPS and MMM), the forage cut after 35 days was significantly higher in crude protein and neutral detergent fiber (NDF) than for the 50-day CI. 

### 3.4. Fixation and Transfer of Atmospheric Nitrogen (N_2_)

#### Estimation of the Contribution of BNF

The values of δ^15^N of the forage and woody material of *L. leucocephala* were not significantly different (*p* < 0.05) between treatments (Table 3). The weed arrowleaf sida (*Sida rhombifolia* L.), assumed to be a non-N_2_-fixing dicotyledonous species, showed a mean ^15^N abundance of +7.51‰ and the *M. maximus* in the grass-alone plots +5.55‰. It was assumed that the ^15^N abundance of the N derived from the soil by the legume would be between these two values and for the calculations the δ^15^N value of the mean of these two values (+6.53‰) was used. The *B* value used was −1.00‰, and the reason for the use of this value is justified in the Discussion.

While the mass of grass forage was higher than that of the Leucaena in both SPS treatments, the concentration of N in the edible legume forage was approximately three times that of the grass. Hence, the total N in the available forage of each component of the association was approximately equal, 76.2 and 72.8 kg N ha^−1^ for the legume and grass, respectively, in the 35-day CI treatment, and 81.5 and 60.9 kg N ha^−1^ in the 50-day CI treatment. The total N in the available forage of SPS systems was 149 and 142.5 for the 35-day and 50-day CI treatments, respectively. For the MMM, the total forage N was far lower, 94.4 and 127.2 kg N ha^−1^, for the 35-day and 50-day CI treatments, respectively.

The estimates of the proportion of N derived from the air (%Ndfa) via BNF were based on the weighted mean ^15^N abundance of the edible foliage and the non-edible woody stems of the Leucaena. The proportions of N derived from BNF were high, at 89% and 95 % for the 35-day CI and the 50-day CI, respectively, and these values were not significantly different (*p* < 0.05—Table 3). The results are presented in Table 3, for both the 35- and 50-day CI treatments (mean of 3 and 2 periods for 35 and 50 cutting intervals respectively). The estimates of N fixed by the legume in the SPS systems over a period of 100 days totalled 77.1 and 80 kg N ha^−1^ for the 35-day CI and the 50-day CI treatments, respectively.

## 4. Discussion

### 4.1. Milk Production 

The average milk yield was 4.7 and 7.4 kg cow^−1^ day^−1^ for the MMM and SPS, respectively, somewhat lower than the milk yields of 9.0 to 9.2 kg cow^−1^ day^−1^ reported by Bacab and Solorio (2011) [15] for SPS on two farms in the State of Michoacán in Mexico. There are many reports of improved milk yield of Leucaena in SPS in Cuba [16] and in the valley of Cauca in Colombia where the introduction of such systems promoted the same production levels as the application of 184 kg N ha^−1^ yr^−1^ as urea on African star grass (*Cynodon plectostachyus* [17]). One farm in this region, Hacienda Lucerna, in the 1990s, produced approximately 9000 L milk yr^−1^ on pastures of African star grass at 3.5 animals ha^−1^ with applications of 200 to 230 kg N as urea ha^−1^ yr^−1^. After the establishment of SPS with 10,000 shrubs of *L. leucocephala* ha^−1^, the pastures now support 4.5 animals ha^−1^ and the farm produces 15,000 L milk yr^−1^ with no N fertilizer [18]. The substitution of such large urea additions by the Leucaena implies inputs of symbiotic BNF to the system of at least 200 kg N ha^−1^ yr^−1^.

One of the main constraints to increasing productivity of cattle grazing low-quality tropical grasses is the limited supply of fermentable nitrogen (NH_3_-N) for efficient microbial growth in the rumen, particularly during the dry season [19,20]. The main supply of NH_3_-N arises from the enzymatic degradation of dietary crude protein in the rumen. Crude protein in Leucaena forage is quickly fermented in the rumen, yielding NH_3_ for microbial protein synthesis. Increased atmospheric nitrogen fixation in the legume forage mass, as demonstrated in the present work, supports the contention that the low environmental impact of SPS derives from the provision of low-cost N sources to the soil, reducing the need for chemical fertilizers and giving rise to production systems with lower GHG emissions [17]. Nitrogen intake in cows grazing SPS is increased by combining high-quality foliage browsed from Leucaena [21] and a tropical grass. This improves the efficiency of N uptake by rumen bacteria, from which amino acids are absorbed from the small intestine, which are, in turn, rendered available for milk protein synthesis in the mammary gland, improving the overall efficiency of N utilization [22]. This is a plausible explanation for the increased milk yield in cows in the SPS. Ruiz-Gonzalez et al. (2013) [23] found that milk production in crossbred cows was significantly increased as a result of the incorporation of *L. leucocephala* (45% of ration DM) into low-quality tropical grass rations. It could also be that the improved rumen environment (higher NH_3_ concentration) in the cows grazing the SPS led to a higher extent of DM degradation in the rumen, thus increasing the intake of metabolisable energy of those cows, resulting in higher milk production. Bottini-Luzardo et al. (2016) [24], found that crossbred cows grazing a SPS with *L. leucocephala* and *C. nlemfuensis* pastures, consumed 34% of total DM as legume forage, resulting in a higher milk yield compared to cows in a grass monoculture.

Additionally, it has been reported that Leucaena foliage contains plant secondary compounds (condensed tannins, saponins) which could decrease protozoa population, leading to reduced ruminal methane emissions [25,26]. Montoya-Flores et al. (2020) [27], found that as the level of Leucaena in ration DM was increased, methane emission of crossbreed heifers was linearly reduced. Similar results were reported by Harrison et al. (2015) [28] in cows grazing Leucaena pastures in Australia, thus contributing to the sustainable intensification of animal production. In this sense, the use of plants rich in secondary compounds, as in SPS, has been proposed as a strategy in the move towards the sustainable intensification of animal production in the tropics [17,29,30].

### 4.2. Forage Yield

With respect to forage yield, it should be noted that total growth periods were different and the cutting/interval (35-day and 50-day CI) treatments were not simultaneous. Nonetheless, computing the growth rate of forage in SPS for the whole period released only a small advantage for 50-day CI. For Leucaena, a 17.6 kg DM ha^−1^ day^−1^ was calculated for the 35-day CI while for 50-day CI it was 19.4 DM ha^−1^ day^−1^. The grass component in SPS behaved similarly, with growth rates of 42.9 and 49.7 kg DM ha^−1^ day^−1^ for 35-day and 50-day CI, respectively. The 10% to 15% growth difference in favor of 50-day CI could be assigned to a slower growth resumption provoked by edaphic and climatic impairment or even by forage overcutting. This latter factor seems to be of minor importance to Leucaena [31], but for Tanzania grass the cutting interval must not exceed the time required for the occurrence of two to three leaves in tillers otherwise plant growth is compromised [32]. Even though tillers were not monitored in this study, the large difference between grass growth rate between 35-day (60.5 kg DM ha^−1^ day^−1^) and 50-day CI (106.9 kg DM ha^−1^ day^−1^) in MMM reinforces that the higher frequency was interfering with the grass growth. On the other hand, the presence of Leucaena compensated the available protein in the pasture. This large difference in grass biomass between 35-day and 50-day CI treatments was also observed for the available N in the pasture, which was 53% greater for the 50-day CI compared to 35-day CI. However, irrespective of cutting/interval, the available N in SPS was similar to the available N in MMM for the 50-day CI, and greater than that in MMM for the 35-day CI. This is one of the benefits of using legume trees as SPS for cattle ranching.

### 4.3. Forage Quality

The crude protein content of the Leucaena forage was approximately three times that of the *M. maximus* and the fiber contents (AFD and NFD) almost half (Table 2). This means that as the total forage DM production of the SPS was similar to that of the MMM, the quality of the available forage diet was far higher which explains the much greater (+57%) milk yield in the SPS system than in the MMM. In the experiment with different cutting intervals, the shorter regrowth period of 35 days promoted a significantly higher protein content, but there were no significant differences in fiber content. Furthermore, there was a significantly higher proportion of legume (31%) in the forage mass when the forages were cut after 35 days of growth compared to 50 days (21%), which implies that the quality of the potentially available diet for grazing animals was considerably higher with a shorter period of re-growth (Figure 4).

### 4.4. Fixation and Transfer of Atmospheric Nitrogen (N_2_)

The values of the ^15^N abundance of the non-edible woody stems of the Leucaena were significantly lower than the edible leaves of the same plants. For intact plants, it is usual to assume that lower values of δ^15^N imply greater proportional contributions of BNF (greater %Ndfa). However, because N isotope fractionation may occur in the translocation, immobilization and retranslocation of N within plant tissues [13,33], the lower values in the woody tissue is unlikely to be related to a higher %Ndfa. For this reason, the proportion of N derived from BNF by the Leucaena plants was calculated based on the weighted mean of the ^15^N abundance of the edible leaves and woody material (Table 3). 

A further problem in calculating the %Ndfa of the legume was the choice of the *B* value (the ^15^N abundance of the N derived from BNF). In the literature, there are several estimations of *B* value for Leucaena. Van Kessel et al. (1994) [34] recorded a *B* value of between +0.6 and −0.2‰, Boddey et al. (2000) [12] reported a value of −0.35‰ and Solorio (2005) [35] −0.25‰. The problem is that some leaves (which should contain N recently fixed and N translocated directly) and weighted means of woody material + leaves showed values down to −0.95‰. This would imply that the plants were obtaining an impossible contribution of over 100% of their N from BNF. We therefore adopted the strategy suggested by Peoples et al. (2002) [36] and Unkovich et al. (2008) [37] and used a *B* value close to the minimum observed value of the legume in the field, −1.00‰. This assumes that some individual plants obtained 100% of their N from BNF.

The concentration of N in the leaves of the *M. maximus* was significantly higher when the grass was integrated into the SPS rather than in monoculture (Table 3). This suggests that there was a transfer of the N fixed by the legume to the grass. Furthermore, the grass in the SPS showed a lower ^15^N abundance than that in the monoculture, further evidence for fixed N transfer to the grass in the SPS. The experiment was installed three years before the samplings, so this transfer may have been from senescent leaves, nodules and roots of the Leucaena, rather than any direct transfer. As Peoples et al. (2015) [38] have explained, because of the many N transformations/translocations in the soil/plant system which incur N isotope fractionation, the N transfer cannot be reliably quantified using ^15^N natural abundance data.

The evaluations of the contributions on BNF (kg N ha^−1^ year) indicate the ability of *L. leucocephala* to fix large amounts of N, between 77 and 80 kg N ha^−1^ in a 50-day period in the rainy season. Other authors have found that Leucaena can obtain up to 285 kg N ha^−1^ year for fields sown at high densities [39,40,41,42]. BNF that occurs in SPS can maintain productive pastures that currently require an annual application of nitrogen fertilizers of 140–200 kg ha^−1^ year [43]. For alternative systems using forage maize, up to 325 kg N ha^−1^ year are being applied. The substitution of the large annual applications of N fertilizer by BNF has very positive economic consequences for producers as well as reducing the possible negative environmental impacts.

The increase in N concentration (crude protein content) of the grass by up to 28% when associated with the legume improves nutritional quality in SPS pastures over that in grass-alone pastures. This increase in N content can be attributed to the transfer of N by *L. leucocephala*. Blair et al. (1990) [44] and Jayasundara et al (1997) [45] reported a 7.6% and 21% N transfer, respectively, of N from the legume to the grass. Increasing the amount and quality of the fodder considerably reduces the time taken for pasture animals to gain weight [46]. In the case of dairy cattle, with SPS it is possible to achieve important increments in milk production without the use of concentrates.

## 5. Conclusions

Crossbred cows grazing a silvopastoral system which incorporated *L. leucocephala* and *M. maximus*, showed higher milk production than cows grazing pastures in a monoculture, probably as a response to the higher supply of microbial crude protein to the small intestine and to the improved overall N balance of the cows. The silvopastoral system produced similar quantities of forage compared to the monoculture pasture system, but with a higher crude protein content. Leucaena in the SPS can assimilate above 80% of its N from biological N_2_ fixation and the results suggested that there was a considerable transfer of fixed N to the grass. Cutting of the legume and grass at 50-day intervals increased fodder production per cut, but when calculated on an equal time basis, it was found that there was a higher quantity and proportion of high-crude protein legume forage with the 35-day cutting interval.

## Figures and Tables

**Table 1:** Forage dry mass (kg DM/ha) and total N accumulation (kg N/ha) by *L. leucocephala* and *M. maximus* in MMM and SPS with two cutting/pruning frequencies (each 35 or 50-day CI).

Cutting	SPS Constituent (kg DM/ha)		Dry Mass (kg DM/ha)		Total N (kg N/ha)	
Interval	*L. leucocephala*	*M. maximus*	SPS	MMM	SPS	MMM
35 days	1846.3	4506.9	6353.2 ^b^	6356.6 ^b^	149.49 ^a^	83.76 ^b^
50 days	1940.2	4967.6	6907.8 ^b^	10,691.2 ^a^	142.47 ^a^	127.86 ^a^

Different letters mean significant differences between pasture types and cutting intervals according to Tukey test at *p* < 0.05.

**Table 2:** Chemical composition of *L. leucocephala* and *M. maximus* in SPS and MMM at two cutting intervals (35 and 50 days).

Specie	Cutting Interval (Days)	Content (%)			
		**DM**	**CP**	**ADF**	**NDF**
*L. leucocephala*	35	24.3 ^b^	29.0 ^a^	24.1	40.3
*L. leucocephala*	50	26.5 ^a^	26.1 ^b^	24.9	42.3
*M. maximus* (associated)	35	22.9	9.9 ^a^	39.8	63.7 ^b^
*M. maximus* (associated)	50	24.6	7.8 ^b^	41.3	68.4 ^a^
*M. maximus* (monoculture)	35	23.8	8.4 ^b^	40.9	68.6 ^a^
*M. maximus* (monoculture)	50	23.4	7.4 ^b^	42.5	69.7 ^a^

Different lower case letters in the same column indicate differences between means by the Tukey test at *p* <0.05.

**Table 3:** ^15^N (δ ^15^N) abundance, N concentration and N accumulation of the two components (forage and woody material) of *L. leucocephala* and the *M. maximus* forage associated in an SPS and in MMM at two cutting intervals (35 and 50 days).

Legumes	Forage			Woody Material			Whole Plant		
	δ ^15^N	%N	Total N	δ ^15^N	%	Total N	Mean ^a^ δ^15^N	Ndfa	Total BNF
	(‰)	%	kg ha^−^^1^	(‰)	N	kg ha^−^^1^	(‰)	%	kg ha^−^^1^
*L. leucocephala* (35)	+0.67	4.38	80.14	−1.11	1.06	10.89	+0.15	89	80.9
*L. leucocephala* (50)	+0.35	4.23	81.57	−1.47	1.05	2.61	+0.33	95	80.0
**Grass**	**Forage**								
	δ ^15^N (‰)	%N	Total N kg ha^−^^1^						
*M. maximus* associated (35)	+3.31 ^a^	1.56 ^a^	69.34						
*M. maximus* associated (50)	+2.19 ^b^	1.23 ^b^	60.90						
*M. maximus* monoculture (35)	+5.49 ^b^	1.33 ^a^	83.76						
*M. maximus* monoculture (50)	+5.62 ^a^	1.18 ^b^	127.27						

^a^ Weighted mean of δ^15^N of edible forage and woody material. Different lower case letters in the same column indicate differences between means by the Tukey test at *p* < 0.05.
